# Supplementary material for: Proton Pump Inhibitors Worsen Colorectal Cancer Outcomes in Patients Treated with Bevacizumab
Source: Cancers (Basel). 2024 Oct 2;16(19):3378. doi: 10.3390/cancers16193378 (PMC11475155; doi:10.3390/cancers16193378)

Supplementary Table 1. Incidence proportion (IP) and event rate of PPI users/Non-users, H2RA users/Non-users, respectively

	**All-cause death**		**CRC-specific death**	
	IP (95% CI)	Event (%)	IP (95% CI)	Event (%)
**Non-users vs PPIs**				
Non-users	337.0 (326.0, 348.2)	3,584 (67.1)	326.5 (315.8, 337.6)	3,473 (65.0)
PPIs	454.1 (425.9, 483.7)	963 (72.1)	441.4 (413.6, 470.6)	936 (70.1)
**Non-users vs H2RAs**				
Non-users	333.2 (324.9, 341.8)	6,034 (69.3)	323.9 (315.7, 332.3)	5,865 (67.4)
H2RAs	374.7 (356.2, 394.1)	1,523 (70.0)	364.7 (346.3, 383.7)	1,482 (68.1)

Supplementary Table 2. The dose-dependent effect of PPIs and H2RAs on overall survival and cancer-specific death

	All-cause death		Cancer-specific death	
	**Adjusted HR (95% CI)**	***p* value**	**Adjusted HR (95% CI)**	***p* value**
**PPIs vs Non-usres**	1.36 (1.27,1.46)	<0.0001	1.36(1.27,1.46)	<0.0001
**Dose response**				
cDDD 0-15	1.18 (1.03, 1.35)	0.0177	1.18 (1.03, 1.36)	0.0158
cDDD 15-32	1.26 (1.11, 1.44)	0.0006	1.27 (1.10, 1.45)	0.0007
cDDD 32-47	1.50 (1.32, 1.70)	<0.0001	1.49 (1.31, 1.69)	<0.0001
cDDD >=47	1.53 (1.34,1.75)	<0.0001	1.54 (1.35, 1.76)	<0.0001
**H2RAs vs Non-users**	1.14 (1.07,1.20)	<0.0001	1.14 (1.07,1.20)	<0.0001
**Dose response**				
cDDD 0-6	1.07 (0.96, 1.19)	0.2383	1.07 (0.96, 1.19)	0.2330
cDDD 6-15	1.19 (1.08, 1.31)	0.0007	1.20 (1.08, 1.32)	0.0005
cDDD 15-32	1.09 (0.98, 1.21)	0.0991	1.09 (0.98, 1.21)	0.1012
cDDD >=32	1.21 (1.09,1.35)	0.0005	1.21 (1.08, 1.35)	0.0007

HR: hazard ratio; CI: confidence interval; PPI: proton pump inhibitor; H_2RA: H_2 receptor antagonist; cDDD: cumulative defined daily dose.

Supplementary Figure 1 Study design flowchart of the cohort study, compared with non-users, PPI users and H2RA users. *The colorectal cancer (CRC) patients under bevacizumab data between 2005 to 2020 in Taiwan was obtained from National Health Insurance Research Database and the Taiwan Cancer Registry. The comorbidities were evaluated based on the year 2005. CRC patients diagnosed between 2006 to 2019 were incorporated. Only newly-diagnosed CRC patients were incorporated into this cohort study. All patients were followed up until year 2020 or death with at least one year follow-up period.

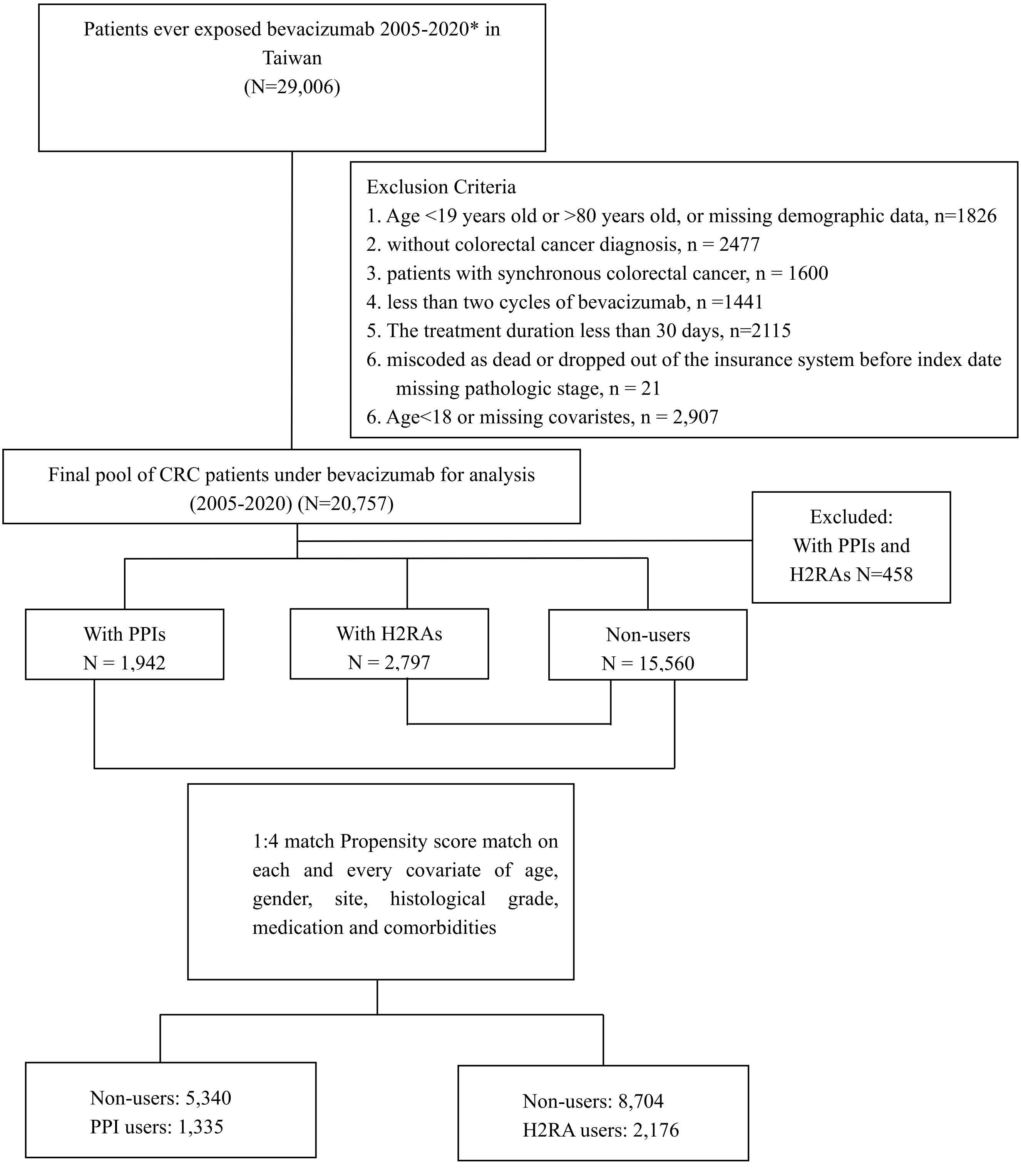

Supplemetary Figure 2. Compared with the patients without ARAs, proton pump inhibitors associated with much more significant reduced overall survival than H2 receptor antagonists in mCRC patients. mCRC: metastatic colorectal cancer; PPI: proton pump inhibitors; H2RA: H2 receptor antagonist.

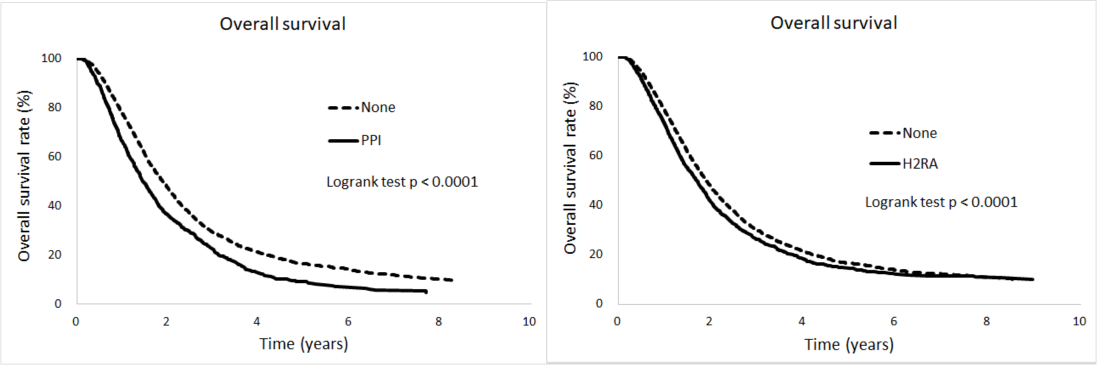

Supplement: Supplementary file 1 [file cancers-16-03378-s001.zip › cancers-3200842-supplementary.pdf]
